# Supplementary material for: Demonstrating an absolute quantum advantage in direct absorption measurement
Source: Sci Rep. 2017 Jul 24;7:6256. doi: 10.1038/s41598-017-06545-w (PMC5524907; doi:10.1038/s41598-017-06545-w)
Supplement: Supplementary file 1 — Supplementary material [file 41598_2017_6545_MOESM1_ESM.pdf]

Supplementary material: Demonstrating an absolute quantum advantage in direct absorption measurement

Paul-Antoine Moreau,[1,2,*] Javier Sabines-Chesterking,[1] Rebecca Whittaker,[1] Siddarth K. Joshi,[1,3] Patrick M. Birchall,[1] Alex McMillan,[1] John G. Rarity,[1] and Jonathan C. F. Matthews[1,†]

[1]*Quantum Engineering Technology Labs, H. H. Wills Physics Laboratory and Department of Electrical and Electronic Engineering, University of Bristol, Merchant Venturers Building, Woodland Road, Bristol BS8 1FD, UK*
[2]*Now at: School of Physics and Astronomy, University of Glasgow, G12 8QQ, UK*
[3]*Now at: Institute for Quantum Optics and Quantum Information (IQOQI) Austrian Academy of Sciences, Boltzmanngasse 3, A-1090 Vienna, Austria*

(Dated: June 12, 2017)

MODEL DERIVATION.

Here we provide the full derivation of an optimised unbiased estimator for the sample absorption α_s that uses both correlated and un-correlated data recorded in the experiment described in the main text. A standard estimator for α_s arises from its definition:

$$\alpha_s = 1 - \eta_s = 1 - \frac{N_1}{\langle N_{1p} \rangle}, \tag{1}$$

where η_s is the transmission efficiency of the sample, N_1 is the number of photons detected on arm 1 after the sample and N_{1p} is the mean number of photons detected on arm 1 without the sample. We can improve upon this estimator by using the fact that we have access to two correlated beams of photons in our experiment. The fluctuation δN_2 of N_2 is defined by :

$$\delta N_2 = N_2 - \langle N_2 \rangle \tag{2}$$

Following [1] we can introduce the corrected estimation N_1' of the photon number after the sample:

$$N_1' = N_1 - k\delta N_2 \tag{3}$$

where k is a correction factor (defined below in eq. (10)). This enables an estimator that uses quantum correlations:

$$\alpha_s' = 1 - \frac{N_1'}{\langle N_{1p} \rangle}. \tag{4}$$

This can be improved further by accounting for classical fluctuations that are recorded by single photon measurements, as given in ref. [1]. We also account for recorded classical fluctuations, however because we intend use in a metrology context we must ensure our estimator is unbiased in order to ultimately provide an accurate estimation of the absorption. We therefore adopt a different approach to the one described in ref. [1].

First we maximise the precision given by our estimator by finding the optimal value of k that minimises the error on the final estimation α_s' of the sample absorption, and for that we have to minimise the variance of N_1'. We express the variance of the photon number for each arm by:

$$\langle \Delta^2 N_i \rangle = N_i + \beta N_i^2 \tag{5}$$

where $i = \{1,2\}$. The first term on the right side of eq. (5) is due to the shot noise limit and the second term is due to classical and deterministic fluctuations (taking into-account super Poissonian environmental noise sources). β is a parameter that quantifies the super-poissonian fluctuations, it is a property of the source and of the noise conditions. Note that the fact that the statistics of each beam on their own is found to be above the shot noise limit is due to two distinct contributions. The first and main contribution is technical noise due to the fact that the pump laser experience itself technical intensity fluctuations and is therefore not a perfect coherent state. The SPDC generated experiences the same fluctuations and the statistics of each beam is as a consequence super-Poissonian. The second contribution is due to the fact that the SPDC statistics is fundamentally thermal because of the existence of stimulated emission. However, it must be noted that for a low number of photons generated per spatio-temporal mode, the thermal statistics tend to a Poissonian distribution of noise because stimulated emission becomes negligible. In our experiment the use of a continuous wave laser means the number of photons per temporal mode is low, and therefore the fundamental thermal contribution, compared to the dominant technical laser fluctuation noise contribution to the super-Poissonian statistics. β is experimentally determined by computing the variance of measured intensity for the individual beams without a sample. We assume symmetric behaviour of the two beams for the sake of simplicity in our model.

Starting from a mean number of photon pairs N and introducing loss η_i in each arm $i = \{1,2\}$ the variance of the measured intensity in terms of photon-number, from eq. (5), is:

$$\langle \Delta^2 N_i \rangle = \eta_i N + \beta \eta_i^2 N^2. \tag{6}$$

Expressing N_1 and N_2 as a function of both the number of generated pairs N and the loss in each channels yields:

$$\begin{aligned} \mathrm{cov}(N_1,N_2) &= \mathrm{cov}(\eta_1\eta_s N, \eta_2 N) = \eta_1\eta_s\eta_2 \langle \Delta^2 N \rangle \\ &= \eta_1\eta_s\eta_2 (N + \beta N^2). \end{aligned} \tag{7}$$

By using equations (3) and (2), this leads to:

$$\langle \Delta^2 N_1' \rangle = \langle \Delta^2 N_1 \rangle + k^2 \langle \Delta^2 N_2 \rangle - 2k\, \mathrm{cov}(N_1,N_2) \tag{8}$$

$$\begin{aligned} &= \eta_1\eta_s N(1+\eta_1\eta_s\beta N) + k^2\eta_2 N(1+\eta_2\beta N) \\ &\quad -2k\eta_1\eta_s\eta_2(N+\beta N^2) \end{aligned} \tag{9}$$

We next search for the value of k that minimises this uncertainty:

$$k=\frac{\eta_1\eta_s(1+\beta N)}{1+\beta N\eta_2} \tag{10}$$

Substituting this value into ref. (4) gives an optimised estimator for α_s taking into account the classical fluctuations through β.

For this estimator to be unbiased, we have to show that the expected value $\langle N_1'\rangle$ of N_1' is equal to the expected value $\langle N_1\rangle$. Which means that we have to explore whether or not $\langle k\delta N_2\rangle=0$. If the expected value of δN_2 tends to zero by definition, then the estimation of η_s, which is dependent on N_1, is not independent from the fluctuations of δN_2.

$$\delta E=\langle k\delta N_2\rangle = k\,\langle\delta N_2\rangle+\mathrm{cov}(k,\delta N_2) \tag{11}$$

$$=\frac{\eta_1(1+\beta N)}{\langle N_{1p}\rangle\,(1+\beta N\eta_2)}\mathrm{cov}(N_1,\delta N_2) \tag{12}$$

where:

$$\mathrm{cov}(N_1,\delta N_2)=\mathrm{cov}(N_1,N_2)=\eta_s\mathrm{cov}(N_{1p},N_2). \tag{13}$$

The last term, $\mathrm{cov}(N_{1p},N_2)$, is the covariance between the two beam's intensities without a sample and is therefore evaluated from the calibration images obtained without sample, since it is a characteristic property of the source and the noise conditions. Using the direct experimental value rather than a model dependant value makes the estimator correction model independent.

We therefore have obtained the following optimised and unbiased estimator that we use to analyse the data in the main text:

$$\alpha_s=1-\frac{N_1'+\delta E}{\langle N_{1p}\rangle}=1-\frac{N_1-k\delta N_2+\delta E}{\langle N_{1p}\rangle} \tag{14}$$

where δE and k are computed independently for each single trial (one image captured by the CCD). This allows the estimation of the sample absorption to be obtained with an optimised estimator each time a measurement is made on the sample, provided only that the source has been previously characterised without the sample present. We obtain k for each trial using the following formula:

$$k=CN_1=\frac{\eta_1(1+\beta N)}{(1+\beta N\eta_2)\,\langle N_{1p}\rangle}N_1 \tag{15}$$

where C is constant from trial to trial. δE is computed by using the same substitution for η_s.

Note that a model that is too simple (defined by eq. (5)) compared to the experimental reality would only make the used estimator non-optimal for a particular experimental circumstance. The model is self-corrected in that the estimator has been insured to be unbiased. Experimentally the following values of the parameters where evaluated: $\beta N=0.32$; $\eta_1=0.59$; $\eta_2=0.65$.

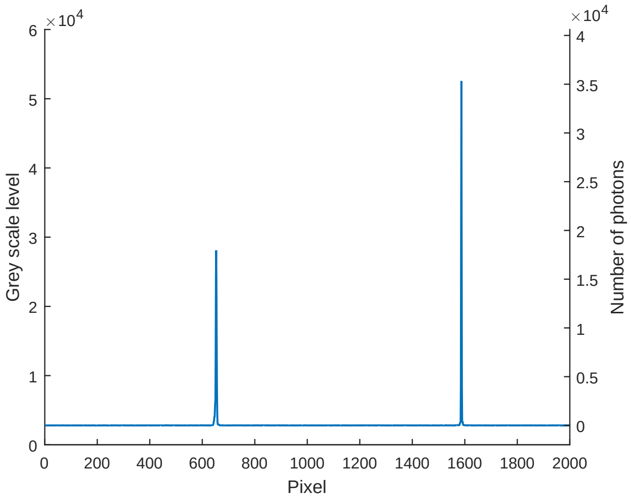

FIG. 1. **Example of raw data extracted from the camera during the acquisition.** this line is obtain by a full vertical binning acquisition of the camera images. The left scale corresponds to the grey scale levels which correspond to the raw data extracted from camera. The right scale correspond to the number of detected photons, obtained after conversion of the scale.

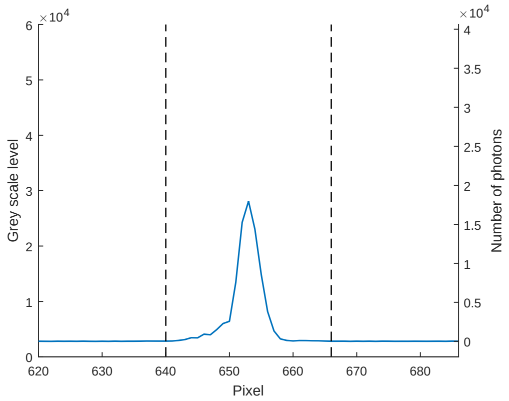

FIG. 2. **Beam profile of the first beam that has gone through the sample.** The black dashed lines correspond to the region of interest inside which the beam intensity is integrated to compute N_1.

COMPARISON TO ANALYSIS REGIME OF REF. [2].

In reporting demonstrations that claim absolute quantum advantage for various technologies, it is illustrative to compare to previous works that report quantum advantage for specific scenarios. In our case, we quantify precision in estimating absorption PPE and we compare our results to the theoretically optimum performance of classical experiments in direct measurement—it is illustrative to repeat this analysis for previous experiments that report sub shot noise absorption imaging for a given detector efficiency. Fig. 4 of [2] gives the ratio of photon pair to classical performance as $R_{\mathrm{SNR}}=1.0455$ at $\sigma=0.45$ which is the mean value of σ in [2]. The data points below $\sigma=0.45$ presented on the figure

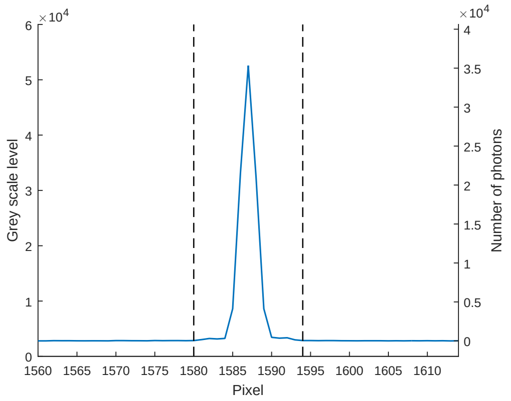

FIG. 3. **Beam profile of the second beam used to correct the fluctuation of beam 1.** The black dashed lines correspond to the region of interest inside which the beam intensity is integrated to compute N_2.

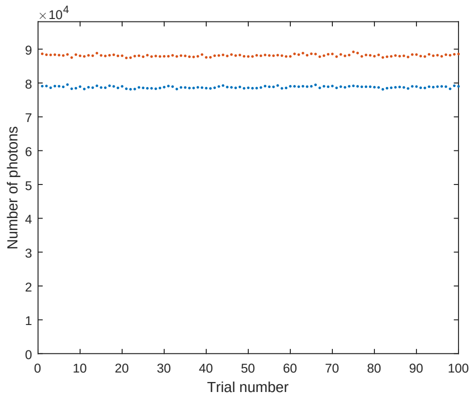

FIG. 4. **Intensities N_1 and N_2, series of 100 measurements.**

are obtained by selecting images that present a better SNR than average and are therefore not statistically representative of the full optical probe used in the demonstration, which is a restriction that we place in our present context in order to compare to potential future classical experiments. We therefore consider the quantum advantage obtained in mean conditions, which means $\sigma = 0.45$ for [2]. As R_{SNR} is defined as an SNR ratio, it is expressed in term of standard deviation. To compare to an advantage in variance we therefore need to take the square of the ratio $\Gamma = R_{\mathrm{SNR}}^2 = 1.0455^2 = 1.093$ which corresponds to a quantum advantage of 9.3%. Since the ratio in [2] is a comparison of the measurement performance with a coherent state with the same number of detected photons, it has to be multiplied by the efficiency of the detector to obtain a ratio comparing to the best possible direct measurement. In [2], it is stated that an efficiency of $\eta = 80\%$ gives $\eta \times \Gamma = 1.093 \times 0.8 \approx 0.87$, so while this result demonstrates sub-shot-noise performance for the given detector efficiency, no absolute quantum advantage is detected. Their demonstration is 13% less accurate that the theoretically optimum classical direct measurement. Furthermore, if we consider the current commercially available CCD technology, which exhibit an efficiency of about $\eta_{\mathrm{now}} = 95\%$[3], then the result reported in [2] can be shown to be less accurate than the best current realistically achievable classical measurement. Numerically, it gives $\frac{\eta}{\eta_{\mathrm{now}}} \times \Gamma = 1.093 \times \frac{0.8}{0.95} \approx 0.92$, i.e. 8% less precise. This supplemental analysis exemplifies the utility of demonstrating absolute quantum advantage, that includes potential future advances in classical technology.

ACQUIRED DATA EXAMPLES.

In this section we show raw data that typical of the data acquired during the reported experimental demonstration. Fig. 1 corresponds to one acquisition with full vertical binning of the 256 vertical pixels of the camera. Two scales are presented. The first corresponds to a grey scale level i.e. the natural output of the camera. The second scale corresponds to the conversion of the acquired data in terms of detected number of photons. Fig. 2 and 3 present the same data, with the region of interest inside which the intensities are integrated to obtain the values N_1 and N_2. Finally, Fig. 4 displays a series of 100 measurement acquired for an absorption of 0.6%.

BEST FITTING IN FIGURE 4 OF THE MAIN TEXT.

The blue fitting in figure 4 of the main text is obtained using the expression:

$$\left\langle \Delta^2 N_1' \right\rangle = \eta_1 \eta_s N(1 + f_a \eta_s + f_b) + k^2 \eta_2 N(1 + f_c) - 2k\eta_1 \eta_s \eta_2 N(1 + f_c) + f_d \tag{16}$$

This correspond to the introduction of 4 fitting parameters (f_a, f_b, f_c, f_d) into equation (9). The fitting parameter f_a corresponds to the deterministic source of super-poissonian noise observed on N_1, f_b corresponds to the non-deterministic source of super-poissonian noise observed on N_1, f_c corresponds to source of super-poissonian noise observed on N_2 and N and finally f_d corresponds to the camera noise.

* Corresponding author: paul-antoine.moreau@glasgow.ac.uk
† Corresponding author: jonathan.matthews@bristol.ac.uk

[1] Tapster, P., Seward, S. & Rarity, J. Sub-shot-noise measurement of modulated absorption using parametric down-conversion. *Physical Review A* **44**, 3266 (1991).

[2] Brida, G., Genovese, M. & Berchera, I. R. Experimental realization of sub-shot-noise quantum imaging. *Nature Photonics* **4**, 227–230 (2010).

[3] Jorden, P. R., Jordan, D., Jerram, P. A., Pratlong, J. & Swindells, I. e2v new ccd and cmos technology developments for astronomical sensors. In *SPIE Astronomical Telescopes+ Instrumentation*, 91540M–91540M (International Society for Optics and Photonics, 2014).
